# Supplementary material for: Green Production of Biomass-Derived Carbon Materials for High-Performance Lithium–Sulfur Batteries
Source: Nanomaterials (Basel). 2023 May 30;13(11):1768. doi: 10.3390/nano13111768 (PMC10255102; doi:10.3390/nano13111768)
Supplement: Supplementary file 1 [file nanomaterials-13-01768-s001.zip › nanomaterials-2350550-supplementary.pdf]

nanomaterials

MDPI

Supplementary Materials

Green Production of Biomass-Derived Carbon Materials for High-Performance Lithium–Sulfur Batteries

Chao Ma [1], Mengmeng Zhang [2], Yi Ding [2], Yan Xue [1,*], Hongju Wang [2], Pengfei Li [2] and Dapeng Wu [2,*]

[1] College of Mechanical and Electrical Engineering, School of 3D Printing, Xinxiang University, Xinxiang 453003, China; mc@xxu.edu.cn
[2] School of Business, Henan Normal University, Xinxiang 453007, China; zhangmengmeng@htu.edu.cn (M.Z.); dingyihnu@163.com (Y.D.); lipengfei@htu.edu.cn (P.L.)
[3] School of Environment, Key Laboratory for Yellow River and Huai River Water Environmental and Pollution Control, Ministry of Education, Collaborative Innovation Center of Henan Province for Green Manufacturing of Fine Chemicals, Henan Normal University, Xinxiang 453007, China; wanghongju@htu.edu.cn (H.W.)
* Correspondence: xueyan6066@163.com (Y.X.); dapengwu@htu.edu.cn (D.W.)

Table S1. Biomass derived carbons for the cathode of LSBs.

Raw Biomass	Method	Surfer and Mass Loading Amount	Surface Area；Dominated Pore and Pore Volume	Initial/final Capacity ；Charge Current	Cycling Stability	Ref.
Bagasse	Sol–gel method; Calcination	75 wt% 1.5mg cm^{-2}	1758.1m^2g^{-1} Mesopores 1.2237 m^3 g^{-1}	863.1/466.9mAhg^{-1} at 1C	800 cycles	[75]
Purple sweet potato	Calcination	67 wt% NG	991 m^2 g^{-1} Micropores/mesopores NG	1035.8/624 mAhg^{-1} at 0.5C	100 cycles	[76]
Peanut shells	Acid-treated Pyrolyzed	68.3 wt% 0.6 mg cm^{-2}	155 m^2 g^{-1} Micropores 0.043 m^3 g^{-1}	778/322 mAhg^{-1} at 0.1A g^{-1}	100 cycles	[77]
Peanut shell	Calcination	57 wt% 0.83 mg cm^{-2}	825.6 m^2 g^{-1} Micropores 1104.3 m^3g^{-1}	1014.6/732 mAhg^{-1} at 0.2C	100 cycles	[78]
Garlic peelings	Calcination Hydrothermal	87.6 wt.% NG	4220 m^2 g^{-1} Micropores 2.02 m^3g^{-1}	1087/NG mAhg^{-1} at 0.1C	400 cycles	[79]
Macadamia nut-shell	Calcination Pyrolyzed	70.1 wt.% NG	3552.7 m^2 g^{-1} Mesopores 2.2 m^3g^{-1}	1252.6/717.3 mAhg^{-1} at 0.2C	200 cycles	[80]
Fallen leaves	Water bath heating Calcination	55 wt % NG	1181.82 m^2 g^{-1} Mesopores 1.0695 m^3g^{-1}	1320/1000 mAhg^{-1} at 0.1C	300 cycles	[81]
Eucommia leaf	Calcination Pyrolyzed	61.70 wt% NG	1724.38 m^2 g^{-1} Micropores, Mesopores and Macropores 1.095 m^3g^{-1}	1336.0/455 mAhg^{-1} at 0.1C	100 cycles	[82]
Balsa waste	Pyrolyzed Calcination	28.92% 1.5 mg cm^{-2}	3,068.40 m^2 g^{-1} Micropores/mesopores 1.37 m^3g^{-1}	1385/925 mAhg^{-1} at 0.1C	100 cycles	[83]

Cattail	Hydrothermal Calcination	87% ~2.37 mg cm^{-2}	2155.81 m^2 g^{-1} Mesopores 1.43 m^3 g^{-1}	1430.6/1216.8 mAhg^{-1} at 0.1C	100 cycles	[84]
Milkweed pappus	Pyrolyzed Calcination	80% 2.5 mg cm^{-2}	1056 m^2 g^{-1} Micropores/mesopores 0.48 m^3 g^{-1}	NG/556 mAhg^{-1} at 0.5 C	200 cycles	[85]
Fluffy catkins	Pyrolyzed Calcination	82.5 wt% NG	793.25 m^2 g^{-1} Mesopores 0.714 m^3 g^{-1}	696.5/ 540 mAhg^{-1} at 0.5 C	500 cycles	[86]
Durian shell	Pyrolyzed Calcination	61.9 wt% NG	2816 m^2 g^{-1} Mesopores 1.6041 m^3 g^{-1}	889.2/402.1 mAhg^{-1} at 0.5C	300 cycles	[87]
Pomelo peel	Pyrolyzed Calcination	53.8 wt% NG	1348 m^2 g^{-1} Micropores/mesopores 0.9123 m^3 g^{-1}	1534.6/717.5mAhg^{-1} at 0.2 C	300 cycles	[88]
Pomelo peel	Freeze-drying Pyrolyzed	91.51% NG	2326.4 m^2 g^{-1} Micropores/mesopores 1.274 m^3 g^{-1}	662.2/527mAhg^{-1} at 1C	300 cycles	[89]
Coffee grounds	Pyrolyzed Hydrothermal Calcination	71.61% NG	2616 m^2 g^{-1} Micropores 1.85 m^3 g^{-1}	1106.7/636.5mAhg^{-1} at 0.2C	200cycles	[90]
Coffee grounds	Pyrolyzed Chemical activation	NG NG	1429 m^2 g^{-1} Micropores 0.54 m^3 g^{-1}	1715/520mAhg^{-1} at 0.5C	1500cycles	[91]
Eggs	Calcination	62.0 wt% NG	693 m^2 g^{-1} Micropores/mesopores NG	769/NG(77%) mAhg^{-1} at 0.2 C	50 cycles	[94]
Ganoderma lucidum	Pyrolyzed Calcination	61.3% NG	2407.5m^2 g^{-1} Micropores/mesopores NG	872.9/631.4mAhg^{-1} at 0.5C	300 cycles	[95]
Acanthopanax senticosus	Hydrothermal Calcination	75.2wt% NG	1415.84 m^2 g^{-1} Micropores 0.7690 m^3 g^{-1}	590.5/540.4 mAhg^{-1} at 0.5C	150 cycles	[96]
Amylose	Pyrolysis Calcination	48 wt % NG	672.6m^2 g^{-1} Micropore 0.32m^3 g^{-1}	1490/798 mAhg^{-1} at 0.1 C	200cycles	[97]
starch	Pyrolysis	81.29 wt% 2.1 mg cm^{-2}	949.85m^2 g^{-1} Mesopores 3.14m^3 g^{-1}	922/683 mAhg^{-1} at 0.5C	100cycles	[98]
Rice Ni	Puffing Pyrolysis	76.1% 2.0mg cm^{-2}	1492.2 m^2 g^{-1} NG NG	1257.2/813.1 mAhg^{-1} at 0.2C	500cycles	[99]
Crude soybeans	Hydrothermal Pyrolysis	80% 5.5 mg cm^{-2}	1500 m^2 g^{-1} Micropores/mesopores 0.7 m^3 g^{-1}	950/460mAhg^{-1} at 0.5C	800cycles	[100]
Cotton stalk	Pyrolysis	67.5% NG	3463.14 m^2 g^{-1} Micropores/mesopores	820/208 mAhg^{-1} at 1 A g^{-1}	200cycles	[108]

116.17m^{2}g^{-1}

Note: NG refers to the data is not given in the reference

Table S2. Biomass derived carbons for the interlayer of LSBs.

Raw Biomass	method	Interlayer types	Surface area; Dominated pore and pore volume	Initial/final capacity ; charge current	Cycling stability	Ref.
Raw eggs sugar	Calcination	coating	451.06 m^2 g^{-1} Micropores/mesopores NG	1198/693 mAhg^{-1} at 0.3 C	100 cycles	[93]
Chlorella	Calcination	coating	1062.16 m^2 g^{-1} Micropores/mesopores NG	1098.24/ 656mAhg^{-1} at 0.5C	600 cycles	[101]
Rice paper plant pith	Hydrothermal Freeze drying Calcination	coating	300.4 m^2 g^{-1} Mesopores 0.186 cm^3 g^{-1}	905.6/ 464.8 mAhg^{-1} at 1C	500 cycles	[102]
Ginkgo Folium	Calcination	coating	1428.1 m^2 g^{-1} Micropores NG	998.7/780.6 mAhg^{-1} at 1C	200 cycles	[103]
Rotten egg albumen	Freeze drying Calcination	coating	10.9 m^2 g^{-1} Mesopores NG	633.8/NG mAhg^{-1} at 1C	400 cycles	[104]
Crab shell	Calcination	coating	1298.2 m^2 g^{-1} Micropores/mesopores 2.10 cm^3 g^{-1}	NG/578 mAhg^{-1} at 1C	500 cycles	[105]
Bamboo	Hydrothermal Calcination	self-support	776.07 m^2 g^{-1} Micropores 0.33 m^3 g^{-1}	907/ 605mAhg^{-1} at 1C	300 cycles	[106]
Luffa sponge	activating Calcination	self-support	3211.2 m^2 g^{-1} Micropores/mesopores 1.72 m^3 g^{-1}	1009.9/719.9 mAhg^{-1} at 2C	500 cycles	[107]

Note: NG refers to the data is not given in the reference
